# Palatable Hyper-Caloric Foods Impact on Neuronal Plasticity

**DOI:** 10.3389/fnbeh.2017.00019

**Published:** 2017-02-14

**Authors:** Jean-Pascal Morin, Luis F. Rodríguez-Durán, Kioko Guzmán-Ramos, Claudia Perez-Cruz, Guillaume Ferreira, Sofia Diaz-Cintra, Gustavo Pacheco-López

**Affiliations:** ^1^Department of Health Sciences, Metropolitan Autonomous University (UAM)Lerma, Mexico; ^2^Institute of Medical Psychology and Behavioral Immunobiology, University Hospital Essen, University of Duisburg-EssenEssen, Germany; ^3^Laboratory of Neurobiology of Learning and Memory, Division of Research and Graduate Studies, Faculty of Psychology, National Autonomous University of Mexico (UNAM)Mexico City, Mexico; ^4^Department of Pharmacology, Center of Research and Advance Studies (CINVESTAV)Mexico City, Mexico; ^5^Laboratory of Nutrition and Integrative Neurobiology, National Institute of Agricultural Research (INRA), UMR 1286Bordeaux, France; ^6^Laboratory of Nutrition and Integrative Neurobiology, Université de BordeauxBordeaux, France; ^7^Institute of Neurobiology, National Autonomous University of Mexico (UNAM)Queretaro, Mexico; ^8^Department of Health Sciences and Technology, Swiss Federal Institute of Technology (ETH) ZurichSchwerzenbach, Switzerland

**Keywords:** obesity, overweight, adiposity, food addiction, indulgent eating, hedonics, neuroinflammation, neural plasticity

## Abstract

Neural plasticity is an intrinsic and essential characteristic of the nervous system that allows animals “self-tuning” to adapt to their environment over their lifetime. Activity-dependent synaptic plasticity in the central nervous system is a form of neural plasticity that underlies learning and memory formation, as well as long-lasting, environmentally-induced maladaptive behaviors, such as drug addiction and overeating of palatable hyper-caloric (PHc) food. In western societies, the abundance of PHc foods has caused a dramatic increase in the incidence of overweight/obesity and related disorders. To this regard, it has been suggested that increased adiposity may be caused at least in part by behavioral changes in the affected individuals that are induced by the chronic consumption of PHc foods; some authors have even drawn attention to the similarity that exists between over-indulgent eating and drug addiction. Long-term misuse of certain dietary components has also been linked to chronic neuroimmune maladaptation that may predispose individuals to neurodegenerative conditions such as Alzheimer’s disease. In this review article, we discuss recent evidence that shows how consumption of PHc food can cause maladaptive neural plasticity that converts short-term ingestive drives into compulsive behaviors. We also discuss the neural mechanisms of how chronic consumption of PHc foods may alter brain function and lead to cognitive impairments, focusing on prenatal, childhood and adolescence as vulnerable neurodevelopmental stages to dietary environmental insults. Finally, we outline a societal agenda for harnessing permissive obesogenic environments.

## Introduction

Given the abundance and omnipresence of palatable hyper-caloric (PHc) foods, overweight and obesity have become a pandemic phenotype in a large portion of the world’s population (WHO, 2016a). Thus, an increased understanding of the underlying causes of obesity is warranted in order to better prevent and treat this growing and global health problem.

Short-term homeostatic control of food intake is essential for animal survival. In addition to this, top-down modulation of homeostatic circuits including palatability and post-prandial rewarding effects modulate food ingestion and seeking behavior (Tulloch et al., 2015). Those drives can support and motivate long-term foraging strategies and planning. In the modern calorie-permissive societies, in which lower energy investments are required to obtain PHc food, those hard-wired capacities, which once evolved to cope with uncertain caloric availability in the wilderness and were evolutionary acquired as adaptive characters, now clearly became maladaptive and do not promote health. Evidence reviewed here suggest that PHc food consumption is self-reinforcing and may further lead to health problems, including cognitive impairments and possibly neurodegenerative diseases that produce a decrease in general wellbeing and productivity. But how eating densely caloric foods can modify brain and behavior in such drastic ways?

**Figure 1:**
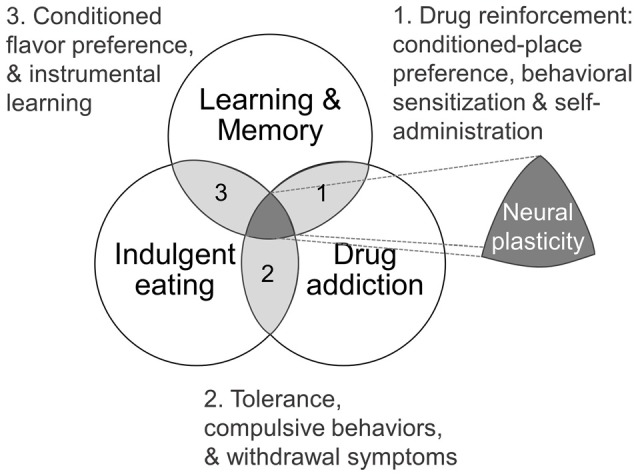
**Theoretical framework as Venn diagram showing intersections of learning and memory, drug addiction and indulgent eating (see text for details)**.

**Figure 2:**
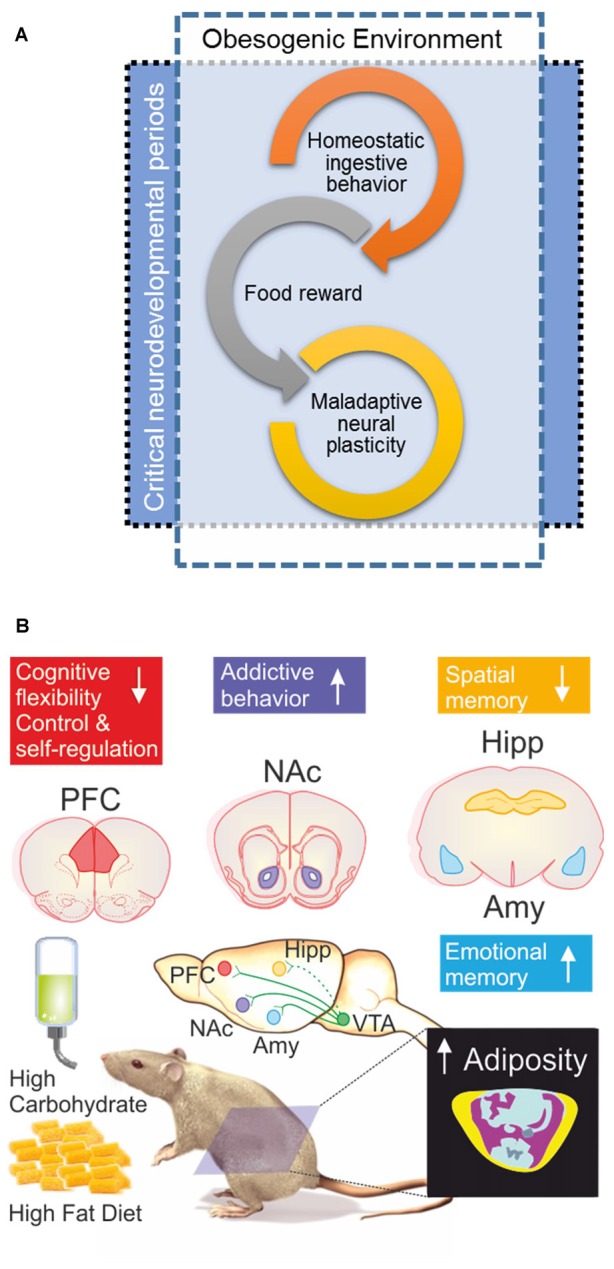
**(A)** When the obesogenic environment overlaps critical neurodevelopmental periods, enhanced maladaptive neural plasticity may be expected; which could eventually lead to uncontrolled ingestive behavior (food addiction). Interplay of food reward and homeostatic ingestive behavior may evolve in wilderness to promote biological fitness under extremely different evolutionary pressures; e.g., scarcity and unpredictable access to food, low dense caloric food, large caloric investments in foraging/hunting. **(B)** Obesogenic environment driven by palatable hyper-caloric (PHc) food can be experimentally modeled in rodents by exposure to high carbohydrate/high fat diet (HFD) resulting in increased adiposity evidenced by body composition analysis by micro-computed tomography; yellow = sub cutaneous fat/pink = visceral fat, blue = lean mass, ultimately causing diet induced obesity (DIO). Experimental evidence documents that exposure to a high carbohydrate/HFD negatively impact on cognitive functions, with increased sensitivity during prenatal, childhood and adolescence neurodevelopmental stages. In particular, hippocampal (Hipp) and pre-frontal cortex (PFC) dependent tasks are negatively impaired; whereas amygdala (Amy) dependent function seems to be enhanced. Cognitive impairments are accompanied (or preceded) by ingestive addictive behaviors driven by the dopaminergic reward system that initiates its projections on the ventral tegmental area (VTA) directly innervating the Amy, PFC, as well as the nucleus accumbens (NAcc; Lisman and Grace, 2005; Russo and Nestler, 2013), the brain structure assessing the hedonic and saliency stimuli properties. It should be remarked that direct projections from VTA to Hipp are on current debate (Takeuchi et al., 2016), thus are depicted with a dash-line. The “reward deficiency syndrome” propose that addiction vulnerability results on from hyporesponsiveness of the midbrain dopaminergic system, leading individuals to seek out and engage in addictive behaviors in order to compensate for underarousal (George et al., 2012), which is in line to the theory of food addiction (Volkow and Wise, 2005; Davis et al., 2011) in particular for PHc food (Ifland et al., 2009; Schulte et al., 2015).

In this review article we will explore the brain plasticity mechanism that contribute to persistent overeating and thus causing overweight/obesity, focusing on the overlap of learning and memory, addictive behaviors and indulgent eating. As well we pinpoint critical neurodevelopmental periods for dietary environmental insults. Graphical summaries are depicted on Figures 1, 2 and key terms definitions can be found as glossary on Table 1.

**Table 1:** **Glossary**.

	
Diet induced obesity (DIO)	Procedure to expose experimental subjects to a hypercaloric diet intervention (e.g., HFD, Western diet).
High fat diet (HFD)	Diet used on pre-clinical experiments usually with at least 45 kcal% from fat (predominately lard). In contrast a control diet contains 10 kcal% from fat.
Homeostatic synaptic scaling	Homeostatic synaptic scaling or simply synaptic scaling is a post-synaptic synaptic plasticity mechanism that changes the global level of postsynaptic AMPA receptors according to a neuron’s activity history.
Long term depression (LTD)	Sustained, use-dependent decrease of the efficiency of a connection between two or more neurons
Long term potentiation (LTP)	Sustained, use-dependent increase of the efficiency of a connection between two or more neurons
Indulgent eating	Indulgent behavior caused by loss of self-control is characterized by time-inconsistent preferences, or a tendency to overweigh short-term rewards relative to more distant ones, and a tendency in the short term to ignore the costs of one’s actions. Thus indulgent eating in some case might be the first step of overeating and other eating behavior disorders.
Metaplasticity	Phenomenon by which the activity history of a given synapse determines its susceptibility to further activity-dependent modification as well as the nature of such modification.
Outcome devaluation	Outcome devaluation occurs when a food reward used during training is devalued by allowing free access to it or by pairing it with an aversive consequence such as gastric malaise.
Overeating/hyperphagia	Is the excess food ingestion in relation to the energy that an organism expends, resulting in overweight/obesity phenotype. It might be related to hypothalamic hyperphagia disorders.
Palatability	Is the hedonic reward provided by foods which often varies relative to the homeostatic satisfaction of nutritional, water, or energy needs.
Pattern completion	Ability to recall an entire memory when presented with a partial sensory cue.
Roux-En-Y gastric bypass surgery	Surgical procedure in which the proximal part of the stomach is cut from the rest. The small intestine is then cut and its distal part is attached to the newly formed pouch below the esophagus, while the proximal part (connected to the larger remaining portion of the stomach) is attached further down. This procedure has been successfully employed in humans to treat morbid obesity.
Synaptic pruning	Widespread process of synapse elimination that occurs during childhood and adolescence, in an experience-dependent fashion.
Synaptic stripping	Removal of dysfunctional synapses by activated microglia.
Western diet (also known as cafeteria diet)	Diet used on pre-clinical experiments where the animal self-selects from palatable, readily available foods including cookies, candy, cheese and processed meats. These foods contain a substantial amount of salt, sugar and fat, which are meant to simulate the human Western diet.

### Neural Plasticity and Addictive Behaviors

One of the most outstanding properties of the nervous system is its ability to modify its structure and function in response to experience, thus allowing individual ontogenic “self-tuning” to particular environmental drivers. The phenomenon of neural plasticity is known to underlie the learning, consolidation and refinement of both adaptive and maladaptive behaviors (Abbott and Nelson, 2000; Citri and Malenka, 2008; Sehgal et al., 2013). At the synaptic level, activity-dependent modifications of the strength or efficacy of synaptic transmission shape the response properties of neural circuits. The versatility and complexity of neural computations is made possible by a huge diversity of cellular plasticity mechanisms (Nelson and Turrigiano, 2008). Those include Hebbian-type plasticity, such as long-term potentiation (LTP) and long-term depression (LTD), as well as homeostatic synaptic scaling and metaplasticity (Pérez-Otaño and Ehlers, 2005).

Some studies have suggested that the development of addictive behaviors share common features with traditional learning models (Figure 1; Jones and Bonci, 2005). For example, N-methyl-D-aspartate (NMDA) receptors blockade, which effectively blocks LTP and LTD in many brain regions (Malenka and Bear, 2004), also prevents many behavioral adaptations normally associated with drug reinforcement, such as conditioned-place preference, behavioral sensitization and self-administration (Mameli and Lüscher, 2011). Furthermore, relapse caused by exposure to cues associated with the drug experience is a major clinical problem that contributes to the persistence of addiction, and its underlying mechanisms are thought to depend at least in part on the phenomenon of pattern completion in the hippocampal CA3 region, which is a hallmark of contextual memory retrieval (Kauer and Malenka, 2007; Kesner et al., 2016). On the other hand, synaptic scaling of α-amino-3-hydroxy-5-methyl-4-isoxazolepropionic acid (AMPA)-receptors surface expression in the nucleus accumbens (NAcc) neurons has been observed with the appearance of addictions (Sun and Wolf, 2009; Tang and Dani, 2009; Reimers et al., 2014). In addition, a single cocaine administration induces metaplasticity in the ventral tegmental area (VTA) through increased synaptic non-GluA2 containing AMPA receptors as well as NR2B containing NMDA receptors, contributing to sensitization upon further exposure, as well as possibly lowering the threshold for further plasticity events in the VTA—NAcc pathway (Creed and Lüscher, 2013). More controversial, however, is the idea that humans can develop “food-dependence” through learning and habit-formation, and that obesity may be seen, at least in some cases, as a clinical manifestation of “food addiction” (Volkow and Wise, 2005; Blumenthal and Gold, 2010; Volkow et al., 2013a; García-García et al., 2014; Carlier et al., 2015). Even though food, as opposed to drugs of abuse, is needed for an organism’s survival, dependence on PHc foods in humans and animal models shares characteristics with drug addiction (Figure 1). These include activation of the mesolimbic dopaminergic system (Blackburn et al., 1986; Hernandez and Hoebel, 1988), the activation of similar brain structures (Robinson et al., 2016), as well as an overlapping symptomatology such as the appearance of tolerance, compulsive behaviors (Johnson and Kenny, 2010; Rossetti et al., 2014) and withdrawal symptoms in relation to PHc food that has been consistently observed in obese individuals (Iemolo et al., 2012; García-García et al., 2014). In this regard, there are many similarities between the eating behavior of some obese individuals and the diagnostic criteria for substances dependence on the *Diagnostic and Statistical Manual of Mental Disorders* (DSM -IV, -5). For instance, both patterns of behavior show signs of: tolerance; withdrawal; substances taken in larger amounts or for longer time than intended; unsuccessful efforts to control usage; a large amount of time spent obtaining, using, or recovering from use of the substance; a neglect of social, occupational, or recreational activities; and continued use despite a recurrent physical or psychological problem caused or exacerbated by the substance (Davis et al., 2011). Following this rationale and aiming to develop a reliable tool for diagnosing food addiction, the DSM-IV criteria for substance dependence have been adapted to create the *Yale Food Addiction Scale* (*YFAS*, Gearhardt et al., 2009, 2016).

Additionally, it is important to recognize that purified and concentrated ingredients used to produce PHc food do resemble the production of addictive drugs that refine cocaine from coca leaf or heroin from poppies (Ifland et al., 2009). There is still scientific debate and no consensus has been reached on the etiological magnitude of food addiction on explaining obesity (Carter et al., 2016), however it is clear by now that in particular PHc foods, like addictive drugs, may produce powerful changes in the brain reward circuitry that we did not evolve for, leading to overconsumption and weight gain. Supporting this view, recent evidence indicates that the addictive effect of food, as for drugs, may be dependent on the rate of its absorption and metabolism; foods reported to be more addictive are rapidly digested and absorbed (Schulte et al., 2015; Criscitelli and Avena, 2016) and are also highly rewarding as we will comment on the next section.

### Reward-Modulated Nutrient Intake

In addition to the homeostatic circuitry that underlie eating (reviewed in Morton et al., 2014), food intake is strongly regulated by hedonic or reward-based signals, which can often override the homeostatic pathways during periods of relative energy abundance by increasing the desire to consume palatable foods (Lutter and Nestler, 2009). Presentation of palatable foods induces potent release of dopamine into the NAcc, originating in the VTA projection, contributing to the motivational and rewarding value of food (Figure 2B). Crucially, the activation of this pathway during meals is related to a loss of control over food intake in some individuals (Stoeckel et al., 2008).

The hedonic component of food intake can be further divided in palatability and post-prandial reward. The palatability subcomponent can be inferred since mammals have innate preference for sweet-flavored solutions over bitter ones independently of their caloric content, and rats learn to prefer a saccharin-sweetened solution over water once it is recognized as safe (Bermúdez-Rattoni, 2004; Yarmolinsky et al., 2009; Drewnowski et al., 2012). Consumption of sucralose, a non-caloric artificial sweetener, induces increases in NAcc dopamine release at levels comparable to sucrose (de Araujo et al., 2008). However, taste palatability alone, independent of its nutritive properties fails to elicit the full rewarding effect of a “good meal”, which integration is dependent upon the summation of relatively independent multisensory “layers of reward”, that include not only taste pleasantness and post-prandial reward, but also visual and olfactory anticipatory cues (de Araujo, 2011).

Post-prandial reward perception is thought to play a central role in the modulation of eating habits (Antoni et al., 2016). In fact, recent evidence has shown that rodents can learn to identify food as rewarding based solely on its caloric content, independently of their taste. For example, ageusic trpm5^−/−^ mice, though initially failing to distinguish between water and a sucrose solution, later develop a preference for sucrose that is indistinguishable from that of wild-types (de Araujo et al., 2008; Simon et al., 2008; Domingos et al., 2011). Pre- and post-absorptive signals from the gut that could alter dopaminergic activity and hence account for the taste-independent rewarding value of sugar are thought to be involved (de Araujo et al., 2012). Indeed, recent evidence has shown that the hormone leptin interfered with the ability of sucrose to produce taste-independent dopaminergic neurons firing. Conversely, other evidences suggest that in addition to its well-established orexygenic effects, the gut peptide ghrelin may have a role in post-prandial reward processing (Müller et al., 2015; Reichelt et al., 2015).

### PHc Food Consumption and Neural Plasticity

Post-prandial reward processing in food consumption involves dopamine efflux in the dorsal striatum (de Araujo et al., 2012). In rodents, this region contains distinct neural circuits that are involved in goal-directed behavior, in the case of the dorsomedial striatum, whereas in habit-based behavior, in the case of the dorsolateral striatum (Figure 2B). Imbalance in these action-control systems is thought to underlie a wide range of neuropsychiatric disorders (Balleine and O’Doherty, 2010). Indeed, there is an extensive overlap between the neural circuits activated by PHc food and drugs of abuse (Kenny, 2011a). In recent years, efforts have been deployed to unveil whether obesity and drug addiction share some common mechanisms, for instance in the long-term modification of reward-seeking behavior (Benton and Young, 2016). In this regard, one crucial question is to ask whether exposure to PHc foods can produce long-term plastic changes in the neural circuitry underlying goal-directed and habit-based behavior? If PHc foods cause some kind of addiction, a shift towards habit-based behavior is expected. This issue was recently addressed by a group of researchers who exposed rats to restricted access to sweetened condensed milk (i.e., PHc food) during 5 weeks and then measured their sensitivity to outcome-devaluation (Furlong et al., 2014). In this case, the task of outcome-devaluation makes use of an instrumental learning paradigm in which animals learn to lever-press for a food reward; once the task is well learned the outcome—the food pellet—is subsequently devalued by allowing free access to it or by pairing it with an aversive consequence such as gastric malaise; so lever-pressing is expected to diminish in animals using a goal-directed strategy. When the task was accomplished via a habit-based strategy instead, the outcome devaluation will not affect the operant response such as pressing a lever. Interestingly, they observed that animals with previous exposure to PHc food, showed greater persistence in lever pressing compared to controls, suggesting that those animals had acquired a habit-based strategy. Also they showed enhanced activation of the dorsolateral striatum, a region involved in habitual behavior. Accordingly, AMPA or dopamine (D)1-receptors antagonism in the dorsolateral striatum rescued behavior to the level of controls. Therefore these results show that a history of consumption of PHc foods may facilitate a shift towards habitual-type control of behavior (Furlong et al., 2014). Importantly, it has recently been shown that behavioral sensitivity to outcome-devaluation is also compromised in obese young men (Horstmann et al., 2015). A study modeling PHc food in rats showed that high fat diet (HFD) exposure from weaning to adulthood reduced instrumental performance and decreased sensitivity to outcome devaluation, suggesting impaired motivation, increased habitual behavior, or both (Tantot et al., 2017). Importantly, these behavioral impairments could be abolished by training adults with a task that reinforces goal-directed behavior (Tantot et al., 2017).

Chronic consumption of PHc food, as it is the case for drugs of abuse, can lead to long-term modifications in the brain circuits involved in reward-seeking behavior (Kenny, 2011b; Volkow et al., 2013b). But food ingestion, as we mentioned, is a complex behavior involving many multisensory reward “layers”. So what characteristic of PHc food is more likely to cause changes in the brain’s circuitry, and ultimately in behavior? To address this issue, a recent study evaluated whether neuronal modifications observed after sustained consumption of PHc correlated with the hedonic value of food, or with its caloric contents (Guegan et al., 2013). For this, they trained mice to lever-press for food rewards that were either normal chow, hypercaloric or palatable isocaloric food and analyzed dendritic spine morphology. In addition, they compared the persistence of food seeking behavior in the three groups of mice once food restriction was relieved. Interestingly, mice trained to obtain isocaloric palatable food showed higher persistence of lever pressing than the two other groups, while having access to food *ad libitum*. Furthermore, non-rewarded lever-press was also higher in mice presented with palatable isocaloric food, suggesting this diet also promoted impulsive-like behavior. Importantly, this behavioral change was not observed in the KO mice for the cannabinoid receptor type 1 (CB1^−/−^), suggesting a role for this endocannabinoid receptors in impulsive food-seeking. When examining dendritic morphology in the three groups, the authors observed that dendritic spine density was increased in the medial prefrontal cortex (PFC) and NAcc shell, regions associated with addictive behavior, in the palatable isocaloric food group, compared to mice that ate hypercaloric food or normal chow. Consistently, this phenomenon was also shown to be dependent on CB1 receptors (Guegan et al., 2013). However, the degree to which neural plasticity mechanisms driven by post-prandial reward interact with those related to learned pleasantness of taste perception remains to be established. It is interesting to note meanwhile, that surgical treatments that have been shown to effectively treat obesity in humans (e.g., bypass surgery) may effectively dampen sweet appetite by interfering with post-prandial striatal dopamine release, as evidenced in a rodent study (Han et al., 2016). In addition, *Roux-En-Y* gastric bypass surgery in rats was shown to alter neural activity in brain regions related to taste perception and reward (Thanos et al., 2015).

As we have reviewed, certain environmental factors and behavior patterns may lead to “food addiction” and ultimately to obesity. Moreover certain lines of evidence suggest that some gene clusters may predispose individuals to both diet induced obesity (DIO) as well as brain inflammation (Heber and Carpenter, 2011). Certain people may therefore be genetically predisposed to absorb fat more efficiently. In addition, DIO by HFD exposure was recently shown to depend on neurotensin, a neuropeptide with significant dopaminergic interactions, and longitudinal studies in humans have shown that pro-neurotensin plasma level is a reliable predictor for the eventual development of obesity (Li et al., 2016). Even though such hereditary view of obesity may slightly downplay the role of behavior and dietary control on obesity, it clearly highlights the fact that a sedentary lifestyle and western diet are at odds with our evolutionary capacity to optimally absorb fats (Bellisari, 2008). In addition, it pinpoints clear pharmacological strategies that may be used in addition to changes in lifestyle and dieting.

### Cognitive Consequences of PHc Food Exposure and Increased Adiposity

It has been reported that PHc foods that lead to obesity are related to a reduced ability to express synaptic plasticity in certain brain areas related to cognition (Dingess et al., 2016; Klein et al., 2016; Tran et al., 2016). For instance, chronic HFD consumption disrupts intracellular cascades involved in synaptic plasticity and insulin signaling/glucose homeostasis (Dutheil et al., 2016) and affects neuronal plasticity-related protein levels (Cai et al., 2016). Nutritional imbalance triggered by this diet eventually impacts glutamate neural pathways, up regulating glial glutamate transporters (GLT-1 and GLAST), down regulating glutamate-degrading enzymes, diminishing basal synaptic transmission and hindering NMDA-induced LTD (Valladolid-Acebes et al., 2012).

Consistently, obesogenic dietary factors, such as simple carbohydrate and saturated fatty acids, have been linked to memory impairments and hippocampal dysfunction (Kanoski, 2012; Sobesky et al., 2014) and evidence suggests that the brain may be particularly vulnerable to obesogenic diets during sensitive neurodevelopmental periods such as pre-natal, infancy and adolescence stages (Figure 2A; Valladolid-Acebes et al., 2013; Noble and Kanoski, 2016; Reichelt, 2016). In rodents, evidence shows that HFD exposure impairs memory of a variety of behavioral test, such as Morris’ water maze, Barnes’ maze, radial arm maze, Y- and T-maze, and novel object recognition (Cordner and Tamashiro, 2015). Interestingly, whereas abundant evidence shows that HFD impairs long-term memory and cognitive flexibility in spatial learning tasks (mainly dependent on hippocampus integrity), some learning processes, such as those that include an anxiogenic or aversive component (amygdala-dependent) may actually be enhanced by such diets (Figure 2B). For instance a recent study found increased emotional memory and amygdala plasticity in rats exposed to HFD from weaning to adulthood, through a mechanism that is dependent on glucocorticoid receptors in the amygdala (Boitard et al., 2015).

Studies in humans have shown that HFD consumption, obesity and metabolic syndrome are associated with poor cognitive performance in children (Bauer et al., 2015; Martin et al., 2016) and adults (Singh-Manoux et al., 2012; Papachristou et al., 2015; Lehtisalo et al., 2016; Yao et al., 2016), and increases risk for development of dementia (Francis and Stevenson, 2013; Freeman et al., 2014). Intake of a HFD that includes mostly omega-6 and saturated fatty acids is associated with worse performance on a cognitive tasks (Kalmijn et al., 1997) and with increased risk for Alzheimer’s disease (Kalmijn et al., 1997; Luchsinger et al., 2002) hypertension and diabetes (Fowler, 2016). In this regards, caloric restriction has been shown to partially revert the HFD effects (Murphy et al., 2014). Individuals adhering to anti-hypertensive diet combined with caloric restriction and exercise show significant improvements in both executive-function memory learning and psychomotor speed when evaluated at 4 months following intervention (Smith et al., 2010). Interestingly, there is strong evidence suggesting that dietary restriction in adult non-human primates has beneficial effects on the preservation of cognitive performance during the course of aging (Colman et al., 2009; Mattison et al., 2012). In addition, a recent meta-analysis suggested that bariatric surgery is generally followed by improved cognitive functions in human patients (Handley et al., 2016), although it should also be warned that under certain circumstances, neuropsychiatric complications, such as increased suicide risk may also occur after this surgical treatment (Peterhänsel et al., 2013; Yen et al., 2014).

New research with animal models has begun to shed light on the neuroinflammatory mechanisms that may underlie the cognitive impairments observed in obese individuals (Castanon et al., 2015). For example, recent evidence in rats showed that fat transplantation produced microglial activation in the hippocampus while lipectomy had opposite effects. The authors went on to show that the cytokine interleukin (IL)-1 positively correlated with adiposity levels as well as cognitive impairments, and IL-1 receptor antagonism rescued the cognitive deficits observed in these animals (Erion et al., 2014; Sobesky et al., 2014). Furthermore, HFD exposure was recently shown to provoke a decrease in hippocampal dendritic spine density as well as synaptic plasticity deficits due to synaptic stripping by microglia, which could be reversed by diet suspension (Hao et al., 2016).

### Prevention and Sensitive Periods to Nutritional Environmental Insults

As is the case for many other diseases, there seems to be critical periods for the development of obesity. Early studies established that gestation, the period between 5 and 7 years of age, and adolescence are critical for the risk of developing long-term obesity (Dietz, 1994), although a more recent longitudinal study suggested that childhood obesity is itself highly dependent on the mother’s diet during pregnancy (Glavin et al., 2014). Studies in rats showed that offspring of dams fed with HFD had higher leptin concentration and glucose intolerance along with increased adiposity (Tamashiro et al., 2009). Similarly in mice, offspring of HFD fed dams show strikingly increased preference for sucrose as well as non-caloric sweetener solution when tested as adults. Interestingly, these mice also show increased sensitivity to cocaine and amphetamine, as well as reduced basal dopamine levels in the striatum and the VTA, which is consistent with higher motivation to obtain food reward (Peleg-Raibstein et al., 2016).

At the neurodevelopmental level, adolescence is characterized by extensive experience-dependent synaptic pruning (Petanjek et al., 2011), as well as changes in gliogenesis and myelination (Fields, 2005; Barbarich-Marsteller et al., 2013; Estes and McAllister, 2016). Moreover, it was recently suggested that blood-brain barrier permeability may be increase by HFD exposure (Kanoski et al., 2010; Hsu and Kanoski, 2014) and is differentially modulated during adolescence (Brenhouse and Schwarz, 2016). Some regions, such as the PFC, which matures up until early adulthood, undergo extensive remodeling and functional plasticity during this period (Reichelt, 2016). In recent years, adolescence has also been established as a critical period for the development of obesity and obesity-related cognitive impairments as some of the underlying neural mechanism are starting to be elucidated (Labouesse et al., 2016; Reichelt, 2016). In a series of experiments, mice were fed HFD during adolescence and later tested in novel location recognition memory, a task that is highly dependent on proper hippocampus function and that is particularly sensitive to manipulations in dorsal CA1 (Assini et al., 2009; Vogel-Ciernia and Wood, 2014). When tested as adults, these mice were less efficient than their control counterparts in this task and this difference was observable even after being switched to food restriction during a 5-week period. In contrast, the same HFD treatment had no effect when administered during adulthood. Intriguingly, this impairment in spatial memory was accompanied by increased neural cell adhesion molecule (NCAM, also known as CD56) accumulation and dendritic spine density increase in the hippocampal CA1 region (Valladolid-Acebes et al., 2013). More recently, adolescent HFD exposure was also shown to alter the levels of the extracellular matrix glycoprotein reelin and impair LTD at PFC synapses (Labouesse et al., 2016). Also it has been observed a diminished neurogenesis and behavioral flexibility in hippocampus-dependent tasks in mice exposed to HFD during adolescence (Boitard et al., 2012). Supporting the notion that PHc food lead to cognitive impairments in particular during vulnerable periods, it has been reported that a HFD supplemented with 10% sucrose was also shown to produce learning and memory impairments in juvenile mice (Xu et al., 2015). More recently, a study demonstrated that rats fed with so-called Western diet (i.e., PHc food) during adolescence had post-traumatic stress responsivity as adults. The study also showed a significant decrease in hippocampal volumes as well as enlarged lateral ventricles in these animals (Kalyan-Masih et al., 2016). Importantly, a promising study showed that by suppressing HFD exposure during adulthood, neurocognitive deterioration seems to be restored in rats even when they were chronically exposed to this diet during adolescence (Boitard et al., 2016).

### Outlook, Living in and Harnessing Permissive Obesogenic Environments

Together, these data provide rationale for particular beneficial effects of early educational/psychosocial interventions, as well as a more aggressive campaigning of warning the effects of PHc food consumption targeting sensitive neurodevelopmental periods; i.e., pregnancy, childhood and adolescence. For instance, it was recently demonstrated that when healthy nutrition is presented as choices that are coherent with adolescent values (such as independence from parents or other figures of authority and freedom from the influence of mass advertising by junk food giants companies), USA eight graders were more likely to stick to a healthy dietary choices (Bryan et al., 2016). Additionally, direct negative monetary incentives were also shown to modulate consumer choice by taxation. For example, in an audacious move trying to control the extreme high prevalence of overweight/obesity, and considering that caloric beverages were major sources of energy among children and adults (Stern et al., 2014), the Mexican government announced the implementation a 10% tax on sugar-sweetened beverages as well as on non-essential food with high caloric density, starting on January 2014. Indeed, a recent analysis confirmed that by December 2014, sales had already dropped by 12% and the data suggested that Mexican consumers were indeed switching to cheaper and healthier alternatives (Colchero et al., 2016).

To increase sales, industrialized food enhances rewarding properties by manipulating salt, sugar, fat, flavors and other food additives to make such foods more like addictive commodities (Cocores and Gold, 2009; Gearhardt et al., 2011; Carter et al., 2016). In the other hand, minimal regulation from governmental health agencies limits food industry and so far there is no public warning about the potential addiction and health problems of PHc food consumption. In this regard, as for other addictive substances like nicotine or alcohol, additional societal support might encourage policy-making bodies to: (a) to start warning about the potential addiction towards PHc food; (b) to regulate PHc food consumption for children, as the first step in modulating adult access to addictive food (Carter et al., 2016); (c) to foster additional research aiming to define the addictive properties of different refined food ingredients/additives as well as its mixture; and (d) to empower consumers by providing clear and straightforward health information in food labels as well as on advertising campaigns.

In summary, recent but indubitable experimental and clinical evidence have documented the deleterious health effects of the permissive obesogenic environment that most western countries are facing, as we have reviewed here, now evidently extending to mental health due to dysregulation in neuronal plasticity (Figure 2A). It is clear that our human physiology did not evolved to face constant and ubiquitous challenges imposed by obesogenic environments, resulting in an overweight/obesity pandemic (WHO, 2016a) that is challenging health systems by imposing unprecedented economic loads (OECD, 2014). Thus it is urgent and necessary to develop comprehensive, long lasting and multidimensional societal agendas to control and revert obesogenic environments by: (a) empowering citizens to take knowledge-based decision and become responsible consumers; (b) protecting consumers in vulnerable stages (i.e., pregnant women, children and adolescents) either by taxation, regulation or bans (WHO, 2016b); and last but not least (c) promoting economic growth based in innovation-driven healthy food alternatives.

## Author Contributions

All authors listed, have made substantial, direct and intellectual contribution to the work, and approved it for publication. In particular: J-PM, LFR-D, GP-L, SD-C, GF, CP-C and KG-R performed literature review. J-PM, GP-L, SD-C and CP-C wrote the manuscript. GP-L designed the figures.

## Conflict of Interest Statement

The authors declare that the research was conducted in the absence of any commercial or financial relationships that could be construed as a potential conflict of interest.
